# Tenofovir-Associated Hypophosphatemic Osteomalacia in an HIV-Infected Patient: A Case Report

**DOI:** 10.7759/cureus.101139

**Published:** 2026-01-09

**Authors:** Ayse Iyiyapici Unubol, Mustafa Unubol

**Affiliations:** 1 Department of Physical Medicine and Rehabilitation, Faculty of Medicine, Aydın Adnan Menderes University, Aydin, TUR; 2 Department of Endocrinology and Metabolism, Private Endocrinology Clinic, Aydın, TUR

**Keywords:** hiv aids, hypophosphatemic osteomalacia, proximal renal tubule, secondary osteoporosis, tenofovir disoproxil fumarate

## Abstract

Hypophosphatemic osteomalacia is a rare metabolic bone disease in adults, most commonly caused by acquired conditions. Clinically, it may present with widespread musculoskeletal pain, proximal muscle weakness, and gait disturbance, often mimicking rheumatologic disorders. Tenofovir disoproxil fumarate, a widely used nucleotide reverse transcriptase inhibitor in the treatment of human immunodeficiency virus infection, may rarely cause hypophosphatemia and osteomalacia due to proximal renal tubular dysfunction.

We report a 33-year-old male patient who presented with progressively worsening diffuse bone and muscle pain, proximal muscle weakness, and difficulty walking over one year and was diagnosed with tenofovir-associated hypophosphatemic osteomalacia. Laboratory evaluation revealed marked hypophosphatemia, phosphaturia, and elevated alkaline phosphatase and parathyroid hormone levels. After discontinuation of tenofovir and initiation of phosphate and active vitamin D supplementation, both clinical symptoms and laboratory abnormalities improved significantly.

This case highlights hypophosphatemic osteomalacia as a potentially overlooked complication of tenofovir therapy.

## Introduction

Hypophosphatemic osteomalacia is a rare metabolic bone disorder in adults, typically resulting from acquired causes and characterized by low serum phosphate levels [1]. Clinically, it presents with diffuse musculoskeletal pain predominantly affecting the lower extremities, proximal myopathy, gait disturbance, and pathological fractures. This clinical picture may mimic various musculoskeletal and rheumatologic diseases, including osteoporosis, fibromyalgia, polymyalgia rheumatica, spondyloarthropathies, and inflammatory myopathies, leading to misdiagnosis or delayed diagnosis [2]. The etiology of hypophosphatemic osteomalacia includes tumor-induced causes, genetic disorders, and medications [3]. Tenofovir disoproxil fumarate is a nucleotide reverse transcriptase inhibitor widely used in the treatment of *human immunodeficiency virus* (HIV) and hepatitis B infections [4]. Rarely, tenofovir may cause proximal renal tubular dysfunction, characterized by impaired tubular phosphate reabsorption, resulting in hypophosphatemia and, in advanced cases, osteomalacia [3,5]. Herein, we report a case of tenofovir-associated hypophosphatemic osteomalacia presenting with clinical features mimicking a rheumatologic disorder.

## Case presentation

A 33-year-old male presented with progressively worsening diffuse bone and muscle pain and difficulty in movement for approximately one year. In recent months, he experienced increasing difficulty rising from a seated position and climbing stairs and reported significant gait impairment. On examination, proximal muscle strength was reduced (Medical Research Council grade 4/5 in hip flexors and shoulder abductors), and the patient had difficulty rising from a seated position and climbing stairs. During this period, he intermittently used non-steroidal anti-inflammatory drugs without significant symptom relief. At the time of the initial examination, the patient did not report using any regular medications. Due to persistent symptoms, he was referred for rheumatologic evaluation with a preliminary diagnosis of spondyloarthropathy. Erythrocyte sedimentation rate and C-reactive protein levels were within normal limits. Antinuclear antibody, rheumatoid factor, and anti-cyclic citrullinated peptide tests were negative. Because of elevated alkaline phosphatase levels, further evaluation for metabolic bone disease was undertaken. Laboratory investigations revealed an alkaline phosphatase level of 437 IU/L (reference range: 40-150) and a bone-specific alkaline phosphatase level of 187 IU/L. Corrected serum calcium was 8.7 mg/dL (reference range: 8.8-10.2), serum phosphate was 0.97 mg/dL (reference range: 2.5-4.5), and parathyroid hormone level was 187 pg/mL (reference range: 18-88). Twenty-four-hour urinary phosphate excretion was 980 mg, indicating significant phosphaturia. Urinary phosphate excretion was quantified by a photometric method. Spot urine analysis showed no glycosuria or proteinuria, and spot urine pH was 6.5. The patient’s serum creatinine level was 0.8 mg/dL, which was within the normal reference range. Bone mineral densitometry showed a lumbar spine (L1-4) Z-score of −5.2 and a femoral neck Z-score of −4.4, consistent with hypophosphatemic osteomalacia. Thoracic computed tomography demonstrated a suspicious hypodense linear defect in the posterior portion of the right second rib, compatible with a nondisplaced fracture. Furthermore, multiple hypodense linear defects consistent with prior fractures were identified in the right fourth, fifth, and seventh ribs and in the left third, fifth, sixth, seventh, and eighth ribs. On three-phase SPECT and whole-body bone scintigraphy, multiple foci of increased radiotracer uptake were identified in the ribs of both hemithoraces, with the most intense uptake in the left tenth rib. Additionally, a focal area of increased uptake was noted in the left iliac wing. The distribution pattern of radiotracer uptake was interpreted as being compatible with a metabolic bone disease. Further etiological evaluation showed that serum fibroblast growth factor-23 (FGF23) levels, measured by enzyme-linked immunosorbent assay (ELISA), were within the normal range. Genetic testing for PHEX mutations associated with late-onset X-linked hypophosphatemic rickets revealed no abnormalities. Detailed history-taking disclosed a previously unreported diagnosis of HIV infection made 2.5 years earlier. The patient had been receiving tenofovir disoproxil fumarate at a dose of 245 mg/day for approximately two years. Baseline metabolic parameters prior to initiation of antiretroviral therapy, including alkaline phosphatase, serum phosphate, parathyroid hormone, and calcium levels, were within the normal range and are now included in Table 1. Based on the clinical presentation and laboratory findings, tenofovir-associated hypophosphatemic osteomalacia was considered the most likely diagnosis. The patient was evaluated for other known adverse effects related to tenofovir therapy, including gastrointestinal symptoms, headache, dizziness, neutropenia, and allergic reactions; none of these were present. There were no clinical or laboratory findings suggestive of generalized Fanconi syndrome or significant renal dysfunction, as urinalysis did not reveal glucosuria or proteinuria, and serum creatinine levels were within the normal range. Tenofovir was discontinued, and the patient was switched to an alternative antiretroviral regimen containing lamivudine and dolutegravir. Neutral phosphate supplementation (250 mg three times daily) and calcitriol (0.25 μg daily) were initiated. Oral cholecalciferol 50.000 IU once weekly was also started. After four weeks of treatment, serum phosphate increased to 1.9 mg/dL, calcium to 9.0 mg/dL, and alkaline phosphatase decreased to 351 IU/L. Due to insufficient improvement in serum phosphate levels, an oral phosphate preparation not available in our country, containing potassium dihydrogen phosphate (602 mg) and sodium hydrogen phosphate dihydrate (360 mg), was obtained from abroad and initiated at a dose of four times daily. At the sixth month of treatment, serum phosphate increased to 2.6 mg/dL, serum calcium stabilized at 9.4 mg/dL, and alkaline phosphatase decreased to 147 IU/L (Table 1). The patient experienced marked improvement in muscle strength, bone pain, and gait, and remained asymptomatic at six-month follow-up.

**Table 1:** Laboratory findings before tenofovir therapy, at presentation, and during follow-up.

Laboratory tests	Reference range	Baseline (before tenofovir)	At presentation	First follow-up (1 month)	Last follow-up (6 months)
Calcium (mg/dL)	8.8-10.2	9	8.7	9.1	9.4
Phosphate (mg/dL)	2.5-4.5	4.2	0.97	1.9	2.6
ALP (U/L)	40-150	132	437	351	147
Albumin (g/L)	35-50	48	48	49	48
Creatinine (mg/dL)	0.67-1.17	0.8	0.8	0.82	0.8
25-hydroxyvitamin D (ng/mL)	30-75	18	10.5	30	35.2
Parathyroid hormone (pg/mL)	18-88	43	187	146	67

## Discussion

This case describes hypophosphatemic osteomalacia associated with tenofovir disoproxil fumarate use. Although tenofovir is an effective and widely prescribed antiretroviral agent, it may rarely lead to clinically significant renal and skeletal complications [3-5]. Tenofovir is eliminated through glomerular filtration and tubular secretion and may cause proximal renal tubular injury. Tenofovir-associated renal damage ranges from subclinical tubular dysfunction to Fanconi syndrome and end-stage renal disease [5]. Long-term tenofovir use has been associated with hypophosphatemia in up to 31% of patients [6], while the incidence of Fanconi syndrome has been reported as approximately 5.5 per 1,000 person-years [7]. Osteomalacia secondary to tenofovir-induced proximal tubular dysfunction remains a rare but serious complication [8]. Although the exact mechanisms of tenofovir disoproxil fumarate-related nephrotoxicity remain incompletely understood, experimental studies suggest that prolonged exposure increases oxidative and nitrosative stress in renal tissue, depletes antioxidant defenses, and induces mitochondrial injury. Accumulation of tenofovir in proximal tubular cells further disrupts mitochondrial DNA synthesis and cellular energy metabolism, promotes apoptosis, and impairs phosphate reabsorption, resulting in renal phosphate wasting, hypophosphatemia, and osteomalacia [9]. Typical laboratory findings in hypophosphatemic osteomalacia include marked hypophosphatemia, normal or mildly decreased serum calcium, increased urinary phosphate excretion, elevated alkaline phosphatase levels, and normal or mildly elevated parathyroid hormone levels. Tenofovir also reduces renal synthesis of active vitamin D, contributing to secondary hyperparathyroidism [10]. In Fanconi syndrome, glycosuria, proteinuria, hypouricemia, and metabolic acidosis may be present [5,7]. In our patient, laboratory findings were consistent with hypophosphatemic osteomalacia, although overt Fanconi syndrome was not evident. Fibroblast growth factor-23 plays a key role in phosphate homeostasis and is clinically important in the differential diagnosis of hypophosphatemic osteomalacia [1]. Normal FGF23 levels in our patient helped exclude tumor-induced and genetic causes. In addition to renal effects, tenofovir has direct adverse effects on bone metabolism. It increases vitamin D receptor expression, reducing the bioavailability of active vitamin D and contributing to secondary hyperparathyroidism [8]. Tenofovir use has also been associated with significant reductions in bone mineral density and increased fracture risk. A large cohort study reported a 12% annual increase in fracture risk associated with tenofovir-containing regimens [11]. The clinical manifestations of tenofovir-induced osteomalacia frequently mimic rheumatologic disorders. Diffuse bone pain, proximal muscle weakness, and gait disturbance are common presenting features. Cases initially misdiagnosed as polymyalgia rheumatica have been reported [2]. Therefore, careful evaluation of serum phosphate levels and alkaline phosphatase is crucial in patients presenting with unexplained musculoskeletal pain. Regular monitoring of renal function and bone-related laboratory parameters may facilitate early recognition and management of tenofovir-associated complications [12]. Discontinuation of the offending agent and appropriate replacement therapy remain the cornerstone of treatment [3,10,13]. In our patient, these measures resulted in significant clinical and biochemical improvement. 

## Conclusions

This case highlights tenofovir-associated hypophosphatemic osteomalacia as an important and potentially overlooked cause of diffuse musculoskeletal pain and functional impairment in patients with HIV. Prolonged exposure to tenofovir may result in significant renal phosphate wasting even in the absence of advanced renal dysfunction. Clinicians should maintain a high index of suspicion for this condition in patients presenting with unexplained bone pain, weakness, or gait disturbances, with serum phosphate levels serving as an important diagnostic clue. Early recognition and timely modification of antiretroviral therapy can lead to marked clinical and biochemical improvement.
